# CD3+CD4-CD8- Double-Negative Lymphocytes Are Increased in the Aqueous Humor of Patients with Retinitis Pigmentosa: Their Possible Role in Mediating Inflammation

**DOI:** 10.3390/ijms252313163

**Published:** 2024-12-07

**Authors:** Daniela Bacherini, Laura Maggi, Francesco Faraldi, Andrea Sodi, Lorenzo Vannozzi, Alessio Mazzoni, Manuela Capone, Gianni Virgili, Giulio Vicini, Benedetto Falsini, Lorenzo Cosmi, Pasquale Viggiano, Stanislao Rizzo, Francesco Annunziato, Fabrizio Giansanti, Francesco Liotta

**Affiliations:** 1 Department of Neurosciences, Psychology, Drug Research and Child Health Eye Clinic, University of Florence, AOU Careggi, 50139 Florence, Italy; daniela.bacherini@gmail.com (D.B.); andreasodi2@gmail.com (A.S.); lorenzo.vannozzi@tin.it (L.V.); gianni.virgili@unifi.it (G.V.); giulio.vicini@gmail.com (G.V.); fabrizio.giansanti@unifi.it (F.G.); 2 Department of Experimental and Clinical Medicine, University of Florence, 50134 Florence, Italy; laura.maggi@unifi.it (L.M.); alessio.mazzoni@unifi.it (A.M.); manuela.capone@unifi.it (M.C.); lorenzo.cosmi@unifi.it (L.C.); francesco.annunziato@unifi.it (F.A.); francesco.liotta@unifi.it (F.L.); 3 Ophthalmology Unit, Surgical Department, A.O. Ordine Mauriziano, 10128 Turin, Italy; 4 UOC Oculistica, Fondazione Policlinico Universitario A. Gemelli IRCCS, 00168 Rome, Italy; benedetto.falsini@policlinicogemelli.it (B.F.); stanislao.rizzo@policlinicogemelli.it (S.R.); 5 Department of Head and Neck and Sensory Organs, Università Cattolica del Sacro Cuore, 00168 Rome, Italy; 6 Department of Translational Biomedicine Neuroscience, University of Bari “Aldo Moro”, 70121 Bari, Italy; pasquale.viggiano@uniba.it; 7 Consiglio Nazionale delle Ricerche, Istituto di Neuroscienze, 56124 Pisa, Italy

**Keywords:** T cells, CD3+CD4-CD8- DN, cytokines, inflammation, retinitis pigmentosa

## Abstract

Recently, evidence has supported a significant role for immune and oxidative-mediated damage underlying the pathogenesis of different types of retinal diseases, including retinitis pigmentosa (RP). Our study aimed to evaluate the presence of immune cells and mediators in patients with RP using flow cytometric analysis of peripheral blood (PB) and aqueous humor (AH) samples. We recruited 12 patients with RP and nine controls undergoing cataract surgery. Flow cytometric analysis of PB and AH samples provided a membrane staining that targeted surface molecules (CD14, CD16, CD19, CD3, CD4, CD8, and CD161) identifying monocytes, natural killer (NK) cells, B cells, T cells, and T subpopulations, respectively. Moreover, lymphocytes were polyclonally stimulated to evaluate cytokine (CK) production at single-cell level. The circulating immune cell distribution was comparable between patients with RP and controls. Conversely, in the AH of controls we could detect no cells, while in the RP AH samples we found infiltrating leukocytes, consisting of T (CD3+), B (CD19+), NK (CD16+CD3-) cells, and monocytes (CD14+). In patients with RP, the frequency of most infiltrating immune cell populations was similar between the AH and PB. However, among T cell subpopulations, the frequency of CD3+CD4+ T cells was significantly lower in the RP AH compared to RP PB, whereas CD3+CD4-CD8- double-negative (DN) T cells were significantly higher in the RP AH compared to RP PB. Cytokine production analysis revealed a trend toward an increased frequency of CD3+CD8-CD161+IFN-ɣ-producing cells and a decreased frequency of CD3+CD8+IL-4-producing cells in the RP AH compared to RP PB. The detection of immune cells, particularly DN T cells, and a Th1-skewed phenotype in RP AH suggests immune-mediated and inflammatory mechanisms in the disease.

## 1. Introduction

Retinitis pigmentosa (RP) refers to a group of inherited retinal dystrophies characterized by the progressive degeneration of rod and cone photoreceptors, leading to visual impairment and eventual blindness [1]. In the Western population, the global prevalence of RP has been estimated to be around 1 in 3000–5000 individuals, making it the most common inherited retinal disease [2]. 

While the primary causes of RP are attributed to genetic defects affecting photoreceptors or the retinal pigment epithelium (RPE), recent research has increasingly highlighted the role of inflammation and oxidative stress in the pathogenesis of various retinal diseases, including diabetic retinopathy [3], age-related macular degeneration [4], and retinal vein occlusion [5]. These findings suggest that inflammation could play a similar role in RP, warranting exploration of anti-inflammatory treatments as potential therapeutic strategies.

Traditionally, the term “retinitis” implied an inflammatory component in RP, with early theories emphasizing inflammation as a key pathogenetic factor. However, despite these initial suggestions, the contribution of inflammation in hereditary retinal dystrophies, such as RP, has not been well defined until recent years. Emerging studies now highlight inflammation as a crucial contributor to the progression of RP. Notably, increased aqueous flare, a sign of blood–retinal barrier (BRB) disruption, has been closely associated with visual function and the extent of retinal degeneration in patients with RP [6,7,8,9,10]. Aqueous flare, which reflects leakage of inflammatory proteins and cells due to a compromised blood–aqueous barrier (BAB), is typically seen in inflammatory ocular disorders, and its presence in RP suggests early involvement of retinal inflammation [11,12]. Microglia, the resident immune cells of the retina, play a central role in retinal inflammatory responses. Gupta et al. demonstrated that microglial activation occurs in RP, particularly in areas of photoreceptor degeneration, where microglia infiltrate degenerative foci and exhibit enlarged amoeboid shapes with cytoplasmic inclusions containing rhodopsin [13]. These activated microglia contribute to retinal inflammation by secreting proinflammatory mediators, phagocytosing damaged cells, and promoting further degeneration. In RP model mice, inhibition of microglial activation or phagocytosis has been shown to reduce photoreceptor degeneration, suggesting that modulating microglial function may mitigate disease progression [14]. Moreover, elevated levels of inflammatory factors in the serum, vitreous, and aqueous humor (AH) of patients with RP further support the involvement of inflammation in the disease [15,16,17,18].

In this study, we aimed to investigate the role of inflammation in the pathogenesis of RP by evaluating potential inflammatory markers in peripheral blood (PB) and AH of patients with RP. By focusing on the AH, we specifically targeted the local inflammatory environment within the eye, which is directly involved in retinal pathology. This approach provides more relevant insights into the immune response at the site of disease development compared to systemic blood or serum analyses. Specifically, we analyzed the cellular profiles and cytokine production in the AH to gain insights into the immune response associated with RP. By examining these inflammatory markers, we seek to better understand how inflammation contributes to RP and to identify possible targets for therapeutic intervention.

## 2. Results

### 2.1. Patient Characteristics 

We included 12 patients with RP and nine controls undergoing cataract surgery. We evaluated the presence of immune cells and mediators in these patients using flow cytometric analysis of PB and AH samples and performed a comparison between the two groups.

The RP group consisted of 12 patients (six males and six females); 14 eyes were considered since 2 patients were operated on in both eyes. A total of 14 AH and PB samples were obtained. 

The mean age was 57.1 years (+/−8.2 years) (range 45–73 years) (variable inheritance, including autosomal dominant, autosomal recessive, and sporadic and two eyes with Usher type 1). The average age of diagnosis was 28.5 years (median 29 years). All patients were at a relatively advanced stage of the disease, with significant visual field impairment at the time of enrollment. For our study we considered 14 eyes, because two patients underwent bilateral cataract surgery.

The control group included nine patients (four males and five females). A total of nine AH and PB samples were obtained. The mean age was 77.11 years (+/−9.8 years) (range 63–92).

Patient characteristics are summarized in Table 1.

Of these 12 patients with RP, four eyes exhibited cystoid macular edema (CME) as a complication of the retinal dystrophy.

In the RP group, the most common indications for cataract surgery were a slow decline in central vision affecting ability to read (11 eyes) and increased glare (3 eyes). 

In the control group, the most common indications for cataract surgery were slow decline in vision (eight eyes) and increased glare (one eye). No intraoperative complications were reported in either group. 

In the control group, the ophthalmologic examination was unremarkable, and mean preoperative BCVA was 20/50 (logMAR 0.4 +/− 0.31). The 1-month postoperative mean BCVA was 20/20 (logMAR 0 +/− 0.10).

In the RP group, mean preoperative BCVA was 20/200 Snellen (logMAR 0.99 +/− 0.51) (range of visual acuity, from hand motions to 20/40). The 1-month postoperative mean BCVA was 20/100 (logMAR 0.73 +/− 0.60) (range of visual acuity, from finger count at 2 feet to 20/25). Mean postoperative vision improved compared with preoperative vision but without a significative difference. The most common postoperative finding was posterior capsular opacity in five eyes (33.3%), and two eyes (13.3%) underwent Neodymium-yttrium-aluminum-garnet (Nd:YAG) laser capsulotomy at a mean of 2.2 months after surgery. 

CME persisted in four eyes (26.6%) of three patients and was treated with a tapering dose of steroids and nonsteroidal anti-inflammatory (NSAID) over 3 months and a topical carbonic anhydrase inhibitor twice daily for 3 months, postoperatively.

### 2.2. Aqueous Humor of Patients with RP-Contained Mononucleated Leukocytes

**Figure 1:**
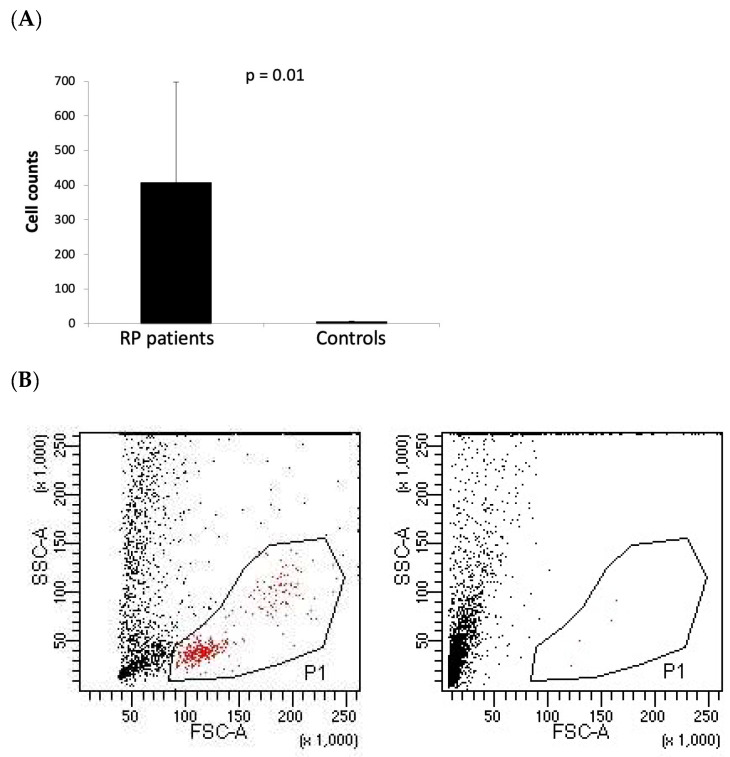
Flow cytometry «counts» of cellular events in aqueous humor (AH) sample. (**A**) AH samples from patients with retinitis pigmentosa (RP) (n = 14) and from controls (n = 9) were analyzed using flow cytometry in order to identify the presence of cells in each sample. The columns represent the mean value and CI of the total number of events recorded in the P1 gate, defined in the plot of physical parameters scatter (FSC/SSC) as classic mononuclear cell gate. (**B**) Flow cytometric dot plots of physical parameters scatter (FSC/SSC) representative of one RP sample (left panel) and one control sample (right panel).

The AH samples from patients with RP (n = 14) and controls (n = 9) were analyzed using flow cytometry. No events corresponding to granulocyte populations, based on physical parameter scatter, were detectable in any of the acquired samples. However, mononuclear cells (MNCs) were identifiable in the AH samples from the RP group. In contrast, the AH samples from the control group were virtually devoid of cells, with a significant difference observed between the groups (*p* = 0.01, Figure 1).

### 2.3. Phenotypical Characteristics of Leukocytes Infiltrating the Aqueous Humor of Patients with RP

PB and AH samples from patients with RP (n = 14) were analyzed using flow cytometry for surface marker expression to permit the identification of T (CD3+) cells, B (CD19+) cells, natural killer (NK) (CD16) cells, monocytes (CD14), and T cell subpopulations (CD3+CD4+CD8, CD3+CD4-CD8+, and CD3CD4-CD8-).

In all AH samples (n = 14) of the patients with RP, we found the presence of infiltrating leukocytes with lympho-monocyte characteristics, mainly consisting of T lymphocytes, including CD4+ and CD8+, but also CD4-CD8- subpopulations, and in lower quantity B lymphocytes, NK cells, and monocytes. 

**Figure 2:**
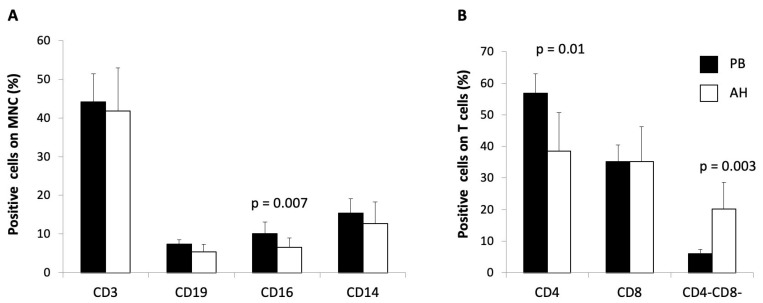
Flow cytometry analysis of immune cell phenotypes at peripheral blood (PB) and aqueous umor (AH) levels in patients with retinitis pigmentosa (n = 14). PB (black columns) and AH (white columns) samples were analyzed using flow cytometry for surface marker expression to permit identification of T cells (CD3+), B cells (CD19+), natural killer cells (CD3–CD16), and monocytes (CD14) in the mononuclear cells (MNCs) (**A**), and of the different T cell subpopulations (CD3+CD4+, CD3+CD8+, and CD3+CD4-CD8-) in the T cells (**B**). Columns represent the mean values + CI of the frequency of the indicated cell population and lymphocyte sub-population.

The frequency of cell subsets present in the AH was different from the PB with a significant reduction of NK cells in the AH (10.9% CI 2.98 in PB vs. 7% CI 2.45 in AH, *p* < 0.01) and of CD4+ T cells (56.9% 6.01 CI SE in PB vs. 38.4% 12.2 CI in AH, *p* < 0.05). Finally, a significant increase in the frequency of CD4-CD8- T cells was evident in the AH compared to the PB (6% 1.36 CI in PB vs. 20.2% 8.5 CI in AH, *p* < 0.01); interestingly, these cells mainly consisted of TCRαβ lymphocytes (≥70%). Data reporting the cell composition of AH and PB samples are shown in Figure 2.

### 2.4. Cytokine Production Profile of T Cells Infiltrating the Aqueous Humor of Patients with RP 

The MNCs in the PB and AH samples from the patients with RP were stained for surface markers and intracellular cytokines after polyclonal stimulation, as described in Material and Methods, for the evaluation of cytokine production at the single-cell level using multiparametric flow cytometry. Lymphocytes were gated on the basis of CD3 expression, permitting the identification of T cells, and on the basis of CD8 expression, permitting the identification of CD8+ T cells (cytotoxic T lymphocytes -CTL-) and CD8− T cells (T-helper lymphocytes -Th-). Due to the few cells obtained from the AH, the cytokine production analysis was performed only on 5 out of 14 samples; the CK production was not evaluated on the CD3+CD4-CD8- subpopulation for this reason.

**Figure 3:**
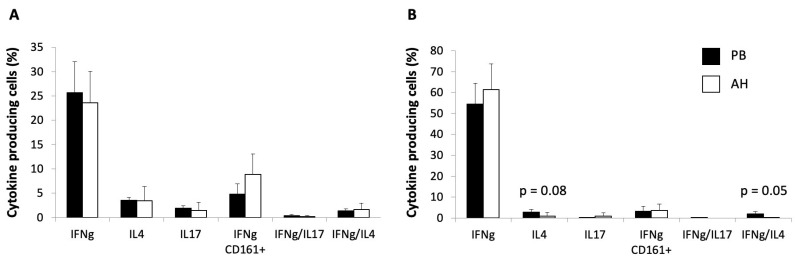
Flow cytometry analysis of intracellular cytokine production. Peripheral blood (PB, black columns) and aqueous humor (AH, white columns) samples from patients with RP (n = 5) were stimulated with phorbol 12-myristate 13-acetate and ionomycin for 6 h, the last four in the presence of Brefeldin A, then fixed and stained for intracellular cytokines associated with surface markers. The flow cytometry analysis was performed on CD3+CD8- (**A**) and CD3+CD8+ (**B**) gated cells. The columns represent the mean values + CI of the frequency of positive cells for the indicated cytokine.

The analysis of cytokine production showed that the T cells deriving from the AH were able to produce a cytokine panel that was similar to that found in the PB; however, there was a slight trend towards an increase in the frequency of CD161+IFN-ɣ+-producing T-helper cells among the CD3+CD8- T cells (4.8% 2.12 CI in PB vs. 7.4% 4.17 CI in AH; *p* = not significant). Moreover, in the group of CD3+CD8+ T cells, we found a significant reduction of the frequency of IFN-ɣ/interleukin(IL)-4-producing cells (1.8% 1.16 CI in PB vs. 0.1% 0.05 CI in AH; *p* = 0.05) and a trend towards a decrease in the frequency of IL-4-producing cells (2.8% 1.37 CI in PB vs. 0.9% 1.7 CI in AH; *p* = 0.08). Data reporting the flow cytometry analysis of intracellular cytokine production are shown in Figure 3.

## 3. Discussion

Retinitis pigmentosa is a group of inherited disorders characterized by progressive peripheral visual field loss, abnormal ERG responses, and variable clinical presentation, severity, age of onset, and progression. Early symptoms can occur in childhood or adolescence and usually consist of night blindness due to loss of the rods. It may lead to a gradual reduction of the visual field and total blindness due to cone involvement in the late stage of the disease [1].

Although several genetic mutations have been identified in patients with RP, the mechanisms through which these mutations lead to photoreceptor apoptosis remain largely unknown.

Rod death is a consequence of genetic mutations, and about 80 RP causative genes have been identified. Conversely, cone degeneration is a late event and thought to result from oxidative damage and inflammation caused by rod loss.

Inflammation is now considered a crucial hallmark of chronic disorders, including cardiovascular disease [19], neurodegenerative diseases [20,21,22], diabetes [23], metabolic syndrome [24], and cancer [25]. Furthermore, recent basic and clinical studies have suggested the importance of chronic inflammation in the pathogenesis of neurodegenerative disorders such as Alzheimer’s disease [26], Parkinson’s disease [27], and degenerative retinal diseases, including RP.

Investigations of inflammatory alterations in patients with RP have been discussed in the recent literature [28], and previous studies have reported the presence of an inflammatory reaction in the eyes of patients with RP, suggesting a potential role of flogosis in RP [29].

Some studies showed the presence of various lymphocyte subsets in vitreous samples obtained from patients with RP [30]. Yoshida et al., by assessing the anterior vitreous of 371 patients with RP with slit-lamp biomicroscopy, found a substantial number of cells in the vitreous cavity in 30% of patients with RP [15]. Moreover, they observed that younger patients with an active disease process were more frequently associated with stronger inflammatory reactions, and patients with RP with more vitreous inflammation had significantly lower visual function. Moreover, the levels of a variety of proinflammatory cytokines and chemokines, including monocyte chemotactic protein-1 (evaluated by a multiplex enzyme-linked immunosorbent assay (ELISA)), increased both in the AH and vitreous fluid of patients with RP compared with controls [15].

These findings underline the relevance of inflammatory processes not only as secondary phenomena but potentially as key contributors to the progression of RP. More studies confirm that the progression of many inherited retinal dystrophies is influenced, among other factors, by the activation of the immune cells and the release of inflammatory molecules such as chemokines and cytokines [31]. A recent study revealed that in patients with RP, intraocular levels of inflammatory cytokines such as IL-2, IL-6, MCP1 (monocyte chemoattractant protein1), and PIGF (placental growth factor) exceeded the levels of serum, indicating intraocular production [16]. A further study confirmed that serum inflammatory cytokines (such as IL-8 and RANTES) levels were significantly increased in patients with RP compared with controls, and the levels of IL-8 were negatively correlated with visual acuity, retinal sensitivity, the central subfield thickness, and ellipsoid zone width [18]. 

In our study, we have investigated the presence and nature of immune cells in the eyes of patients with RP using flow cytometry; we also evaluated the functional property of these cells by performing the cytokine profile at the single-cell level. 

We have detected in RP AH samples the presence of infiltrating leukocytes with lympho-monocyte characteristics, consisting of T lymphocytes (CD3+, both CD4+ and CD8+, and CD4-CD8-), B lymphocytes (CD19+), NK cells (CD16+CD3-), and monocytes (CD14+), while no cells were present in the AH of the controls. Of note, red blood cells were virtually absent in all the samples evaluated, thus excluding blood contamination. 

These results provide direct evidence of a distinct inflammatory cell profile in the AH of patients with RP, confirming previous observations of immune activation in RP [32]. 

Interestingly, the cell composition of AH was limited to MNCs; moreover, lymphocyte composition was different from that found in the blood sample of the same patients, with a significant reduction in NK cells and CD4+ Th cells, and with a significant increase in CD3+CD4-CD8- T cells, mainly made up of TCRαβ lymphocytes.

These data suggest the hypothesis of an impairment of the BRB allowing the migration of circulating inflammatory cells toward the intraocular fluids. 

We can speculate that these cells migrate from the bloodstream into the eye, recruited by an as yet unknown pathologic process, which could be due either to cell damage of degenerative origin, such as rod death, followed by antigen spreading and consequent MNC infiltration, or could be due to a primary autoimmune insult. 

In our analysis, the frequency of NK cells was significantly inferior in the AH compared to the PB, rendering improbable a possible role of these cells in the local inflammatory process. The eye is characterized by immune privilege, and the absence of a lymphatic system is important to isolate it from the immune system and to avoid possible damage due to immune reaction. Alteration of this physiologic condition leads to immune-mediated disease of the eye involving both innate and adaptive immune response. Moreover, the corneal endothelium and retina do not express the major histocompatibility complex (MHC) class I molecules and are a potential target of cytotoxic activity by NK cells; this could represent the starting point or the progression of an inflammatory condition. It has been shown that the AH contains a wide range of soluble factors inhibiting immune response, in particular NK-mediated cytotoxicity, the main one being represented by TGF-b [32,33]. We could speculate that the ocular microenviroment contributes to reducing the frequency of NK cells in order to maintain tissue protection and support the immunological privilege. 

Similarly, the frequency of the CD3+CD4+ T cell (T helper lymphocytes) subset was significantly reduced in the AH compared to the PB with a very strong increase in the CD3+CD4-CD8- T cell subpopulation. 

Double-negative (DN) CD4-CD8- T cells, which compose approximately 1–3% of total T cells in healthy human peripheral blood mononuclear cells [34], have been recently described as a cell population of circulating mature T cells of unclear origin. According to emerging data, CD3+CD4-CD8- DN T cells, a rare subset of peripheral T cells, may play diverse roles in the pathogenesis of autoimmune and inflammatory systemic diseases, including systemic lupus erythematosus, Sjögren’s syndrome, and psoriasis, by inducing systemic inflammation and tissue damage [35]. While traditionally less studied than CD4+ and CD8+ T cells, DN T cells are now recognized for their multifunctionality in immune responses, with regulatory DN T cells demonstrating therapeutic potential in autoimmune diseases and helper-like DN T cells exhibiting context-dependent roles in inflammation, viral infections, and autoimmunity. Additionally, recent insights suggest that DN T cells may also contribute to malignancies and hold promise as therapeutic tools in cancer, underscoring their complex and multifaceted contributions to immune regulation and disease progression [36]. In the context of retinal degenerative diseases such as RP, our findings suggest that DN T cells may contribute to chronic inflammation and tissue damage. Their presence in the AH at higher frequencies compared to PB highlights a potential role in local immune responses associated with RP.

DN T cells neither express γδTCR nor NK cell markers; they do not have regulatory T cell markers such as FOXP3, CD25, or CTLA-4 [35,37]. Moreover, these cells show a reduced/absent expression of the co-stimulatory molecule CD28 [37]. This phenotype, characterized by reduced CD28 expression, is typically associated with antigen-experienced and differentiated T cells. Such features align with the hypothesis that DN T cells in RP are recruited and activated in response to chronic antigenic stimulation following photoreceptor degeneration. Of note, CD28-deficient T cells are generally considered antigen-experienced and differentiated elements; accordingly, DN T cells are CCR7+CD45RA-, thus they display phenotypic characteristics of terminally differentiated and “exhausted” T cells [35]. The accumulation of these cells in the AH suggests that they may play a mechanistic role in RP progression through persistent immune activation and potential interaction with retinal antigens exposed during degeneration. While our data support a link between DN T cells and RP, further research is needed to delineate whether these cells contribute directly to retinal damage or represent a secondary response to ongoing photoreceptor loss and inflammation. To our knowledge, this is the first description of DN CD4-CD8- T cells in patients with RP.

With regard to CK production by in vitro-stimulated T cells, we obtained only preliminary data showing some interesting trends, with no statistical significance due to the low number of experiments (n = 5) in which the cell number was sufficient to perform the cytokine stimulation assay. For the same reason, the CK measurement was performed on CD3+CD8- and CD3+CD8+ T cells, but not on the less frequent CD3+CD4-CD8- T cell subpopulation. In particular, we found a slight increase in the frequency of CD161+CD4+ T cells producing IFN-ɣ in the AH, as compared with circulating lymphocytes, and a trend toward a decrease in CD3+CD8+ T cells producing IL-4 and double-producing IFN-ɣ plus IL-4. These data together are in favor of a cytotoxic cell profile in the AH that is consistent with a pathogenetic role for these infiltrating lymphocytes. Of note, the CD161+CD4+IFN-ɣ+ T cell population has already been described, by our group and others, as a Th17-derived phenotype involved in the tissue damage during various autoimmune/inflammatory diseases [38,39]. The Th1-oriented response leads to monocyte/macrophage activation, with subsequent oxidative stress of the tissue.

In general, all our data agree with the hypothesis of a pathogenetic role for immune cells, and therefore inflammation, in RP. The initial mechanism, which may lead to ocular inflammation in patients with RP is not clear at the moment, but some hypotheses may be made.

First, most dystrophic and degenerative diseases are accompanied by low-grade inflammation. It is well known that in RP increased retinal lipofuscin fluorophores may determine damage, disturbed polarity, death of the RPE, and apoptosis of photoreceptors [40]. In response to this stimulation, the RPE synthesises, and releases a wide variety of inflammatory molecules such as cytokines and chemokines [41], with microglial activation contributing to a proinflammatory phenotype. These events may promote the recruitment of inflammatory cells that leak into the vitreous and may reach the AH, as there is no barrier separating the posterior from the anterior segment [42]. Secondly, as the BRB breakdown occurs both in retinal vessels and in the RPE [43,44], even the blood–aqueous barrier (BAB) may be affected, leading to an increased number of inflammatory components in the AH. 

Neuroinflammation is currently considered an early event in the pathophysiology of many neurodegenerative disorders: despite its essential role in protecting tissue at the beginning of disease, the continuous presence of proinflammatory stimuli eventually induces cellular damage [22,45,46]. It is generally accepted that astrocytes and microglia are the cells that in the central nervous system play a critical role in the neuroinflammation preceding neurodegenerative diseases. In this scenario, activated microglia contribute to the release of different inflammatory mediators, including cytokines, chemokines, reactive oxygen species (ROS), and nitric oxide (NO), all of them participating in maintaining an oxidative stress and chronic neuroinflammatory milieu that ultimately could be responsible for oxidative stress and neurotoxic damage in the central nervous system [47].

In this context, hyperactivation of microglial cells has been shown to play an important role in photoreceptor neurodegeneration in animal models of RP [48]. A recent study using live-cell imaging in the rd10 mouse model of RP has identified that the initiation of rod degeneration is accompanied by early infiltration of microglia, upregulation of phagocytic molecules in microglia, and presentation of “eat-me” signals on mutated rods. During the early stages of the disease, the microglia are able to migrate, interact with, and phagocytize non-apoptotic photoreceptors, after which they become hyperactivated and promote the loss of non- and apoptotic photoreceptors [14]. The authors of this study propose that primary microglial phagocytosis may be a contributing mechanism underlying cell death in RP and microglia as a potential cellular target for therapy. Since activated microglia release neurotoxic and/or inflammatory mediators, including TNF-α, interleukin-1β (IL-1β), IL-6, and glutamate, and increase the expression of inducible nitric oxide synthase (iNOS), all of them exacerbating the death of retinal neurons, this could contribute to the breakdown of the BRB. The outer BRB expresses immunoregulatory molecules that inhibit lymphocyte activation, while the RPE of the BRB secretes immunomodulatory mediators that control the immune and inflammatory responses within the eye/into the AH [49]. Therefore, in physiological conditions, PB immune cells are not able to enter the retina to mediate endogenous insults; instead, after BRB breakdown, a recruitment of inflammatory cells from the bloodstream occurs, attracted by chemokines probably produced by activated microglial cells, leading to an increased number of inflammatory components in the AH. A simplified hypothesis is that the underlying genetic defect is the trigger of rod degeneration, which drives a concomitant microglial activation and local increase in production of inflammatory species. In the long run, inflammation may become detrimental for all the cells, and particularly for the cones, known to be especially vulnerable because of their elevated metabolism and high degree of specialization. This would concur with their degeneration, thus conferring to a secondary process (inflammation) a major role in cone vision loss, which is the most severe consequence of RP for humans. The importance of research aimed at understanding the function of the different inflammatory processes in the retina, and especially the contribution of the microglial-mediated neuroinflammation that precedes neurodegeneration, could provide useful knowledge to implement new therapies. Potentially, primary microglial phagocytosis could be a potential cellular target for therapy. However, more research is needed to detect further molecular mechanisms involved in microglial activation in degenerative retinal diseases.

The findings of our study support the presence of an intraocular inflammatory microenvironment in RP, evidenced by infiltrating lymphomononuclear cells in the AH. However, it remains unclear whether these immune responses represent a primary driver of RP progression or a secondary/tertiary consequence of photoreceptor degeneration. Given the long-standing disease duration in our cohort, it is plausible that the observed immune responses are a result of chronic rod and cone degeneration. This aligns with the hypothesis that rod death due to genetic mutations triggers secondary processes, such as antigen spreading, recruitment of inflammatory cells, and BRB breakdown.

Importantly, while our data suggest the presence of chronic inflammation, they do not clarify whether the immune responses in RP represent acute or delayed effects. The predominance of DN T cells (CD3+CD4-CD8-) in the AH compared to PB, coupled with the reduced frequency of NK cells and CD4+ T helper cells, points toward an ongoing adaptive immune response rather than an acute inflammatory event. DN T cells have been implicated in autoimmune and chronic inflammatory conditions, suggesting that their accumulation in RP may be a delayed response to sustained retinal degeneration and microglial activation.

Although the inflammatory alterations observed in this study could contribute to disease progression, we cannot definitively conclude that they are underlying factors for RP development. The use of a cross-sectional design and the absence of a direct correlation between immune cell profiles and the patients’ genotypes or phenotypes, as well as the relatively advanced disease stage of our cohort, limits our ability to determine causality. It is possible that the observed immune activity reflects a tertiary process exacerbating cone degeneration, which is a key determinant of functional vision loss in RP.

To distinguish between primary and secondary inflammatory processes, future studies should consider enrolling patients at earlier stages of RP. Moreover, longitudinal analyses could help identify whether specific inflammatory markers, such as cytokines or immune cell subsets, precede or follow photoreceptor degeneration.

Unfortunately, in our study we could not find any correlation in RP between the humoral and cellular immunological alterations and the genotype and clinical phenotype. We can hypothesize that these flogistic abnormalities could be more significant in patients with macular edema (commonly considered of flogistic origin), but at present we have no conclusive data on this issue.

We are aware of some limitations of our study, in addition to those discussed above, resulting from its cross-sectional design. First, the sample size is relatively small, but we must consider that RP is a rare disease and that AH samples can be obtained only during surgery. Moreover, our groups are not age-matched, and the controls are significantly older than the patients with RP. However, this difference should not interfere with the report on inflammatory cells in the AH of patients with RP, as it has been ascertained in the literature that increasing age is associated with increasing levels of intraocular cytokines and probably of inflammatory cells [16].

Our findings highlight the need for validation studies in longitudinal, larger cohorts and earlier disease stages to explore their diagnostic utility, role in disease monitoring, and potential to guide therapeutic strategies targeting inflammation in RP, while acknowledging the pilot and exploratory nature of this study.

Given the critical involvement of lymphocytes in the pathogenesis of several neurodegenerative diseases, such as multiple sclerosis, elucidating their role in RP could uncover a shared lymphocyte signature across neurodegenerative conditions. This insight may pave the way for innovative therapeutic approaches targeting immune-mediated mechanisms in RP and other neurodegenerative diseases.

## 4. Materials and Methods 

### 4.1. Study Design

This interventional study recruited patients with typical RP undergoing cataract surgery referred to the Hereditary Retinal Degenerations Referring Center of the Eye Clinic, University of Florence, AOU Careggi, Florence, Italy. 

The study adhered to the principles outlined in the current version of the Declaration of Helsinki (52nd WMA General Assembly, Edinburgh, Scotland, October 2000), and written informed consent was obtained from all patients prior to participation in the study. Approval was granted by the Institutional Review Board/Ethics Committee of AOU Careggi.

Inclusion criteria for the RP phenotype were as follows: history of nyctalopia; peripheral visual field constriction; ophthalmoscopic findings: bone spicule–like pigment clumping, vessel attenuation, waxy pallor of the optic disc; marked reduced or non-recordable a- and b-wave amplitudes on electroretinogram testing. All patients carried genetic mutations. The control group consisted of 9 healthy volunteers with age-related cataracts, unrelated to the patients with RP included in the study. All patients with RP underwent a standard ophthalmologic examination, fundus photography, an optical coherence tomography (OCT) scan (Nidek, RS3000 Advance 2, Gamagori, Japan), and fluorescein angiography (Zeiss Retinography with Image Processing Software Visupac (version 4.5), Carl Zeiss, Dublin, CA, USA). Baseline best-corrected visual acuity (BCVA) was measured for all patients using Early Treatment of Diabetic Retinopathy Study (ETDRS) charts and then converted into a logarithm of the minimum angle of resolution (logMAR) for statistical analysis. Electrophysiological testing (ERG) was performed on patients with RP using the Electrophysiological Diagnostic Unit Retimax (Roland Consult, Brandenburg, Germany), following ISCEV guidelines [50]. The controls underwent a standard ophthalmological examination and OCT.

Exclusion criteria included myopia > 8 diopters, hyperopia > 5 diopters, ocular disorders other than RP and significant systemic diseases, a history of inflammation-associated conditions, such as uveitis or proliferative diabetic retinopathy, or a history of intraocular surgery. Information was also collected on relevant past medical history, smoking history, ocular history, use of medication, vitamin, or dietary supplementation. All patients underwent standard phacoemulsification with intraocular lens (IOL) insertion for cataract removal. The cataract surgery was performed by the same surgeon (L.V.) who conducted standard phacoemulsification and IOL implantation through a superior corneal incision in all cases. Yellow-colored acrylic foldable intraocular lenses were used in all cases. The microscope light was reduced to 60% of normal intensity during the surgery, and the light was covered between different cataract steps to minimize photic exposure. Standard post-cataract medication included topical antibiotic drops (moxifloxacin hydrochloride 0.5%) for 1 week, a tapering dose of topical steroids (prednisolone acetate 1%), and a topical NSAID agent (nepafenac 0.1%) 3 times daily for 1 month. No surgery-related complications were observed during the procedure or the 3-month follow-up period.

### 4.2. Sample Collection

AH samples (150–200 µL) were collected during the beginning of a cataract extraction just before capsulorexis, while replacing the AH with a viscoelastic chamber maintainer, avoiding any contact with the intraocular tissues. Immediately after collection, the first operator carried out the rest of the surgical procedure, while a second operator handled the sample. Samples were immediately placed in sterile 1.5 mL polypropylene tubes and carried to laboratory rooms in the closed building. Samples with obvious bleeding were excluded. PB samples were paired with the AH of the patients with RP and controls. All samples were maintained at room temperature until the analysis procedure that was performed at 1 and half hours from the sample collection.

Flow cytometric analyses were performed in the Department of Experimental and Clinical Medicine, University of Florence, Italy. The PB was layered on a density gradient to obtain mononuclear cells (PBMNC) for a phenotypic evaluation by flow cytometric analysis; the sample of AH was centrifuged at 2500 rpm for 8 min: the supernatant was removed, and the cells were resuspended to obtain phenotypic assessment by flow cytometric analysis. 

Flow cytometric analysis of both cellular samples (PB and AH) provided a membrane staining that targeted surface molecules and an intracytoplasmic staining that targeted cytokines. The evaluation of the surface molecules (namely CD14, CD16, CD19, CD3, CD4, CD8, and CD161) permitted the identification of monocytes, NK cells, B cells, T cells, and T cell subpopulations. The study of cytokine production was performed to identify the different types of T-helper cells (Th1, Th2, and Th17) in the different samples. 

### 4.3. Reagents

The medium used for cell cultures was RPMI 1640 (Seromed, Berlin, Germany), supplemented with 2 mM of L-glutamine, 1% non-essential amino acids, 1% pyruvate, and 2 × 10^−5^ M 2-mercaptoethanol (all from Gibco Laboratories, Grand Island, NY, USA). Additionally, 10% fetal calf serum (Euroclone, Pero, Italy) was added to the medium. Fluorochrome-conjugated monoclonal antibodies (mAbs) and isotype-matched control mAbs were sourced from BD Biosciences (Mountain View, CA, USA) or Miltenyi Biotec (Bergisch Gladbach, Germany). For stimulation, phorbol 12-myristate 13-acetate, ionomycin, brefeldin A, formaldehyde, and saponin were from Sigma Aldrich Co. (St. Louis, MO, USA). The method for obtaining MNCs involved centrifugation of PB samples on a Ficoll–Hypaque gradient. For AH samples, cell suspensions were prepared by centrifugation, and the resulting pellet was resuspended in 0.5 mL of RPMI medium supplemented with 10% FCS. This protocol ensured the proper isolation and preparation of cells for subsequent analysis.

### 4.4. Surface Markers Evaluation

To evaluate cell surface markers by flow cytometry, 0.5 × 10^6^ MNCs from PB and one-third of the total cells recovered from the AH were first saturated with rabbit IgG to block nonspecific binding. The cells were then incubated for 15 min on ice in the dark with specific fluorochrome-conjugated monoclonal antibodies (mAbs), as listed in Table 2. After incubation, the cells were washed to remove unbound antibodies. Flow cytometry analysis was performed using a BDLSR II instrument with FACSDiva software (version 9.0), and a total of 10^4^ events were acquired for each PB sample. For the AH samples, all events in each sample were recorded to ensure a comprehensive analysis.

### 4.5. Intracellular Cytokine Evaluation

To perform flow cytometry analysis of intracellular cytokines, 1 × 10^6^ MNCs from PB and two-thirds of the total cells recovered from the AH were stimulated with phorbol 12-myristate 13-acetate (PMA, 10 ng/mL) and ionomycin (1 µM) for 6 h, with the last 4 h in the presence of Brefeldin A (5 µg/mL), in RPMI supplemented with 10% fetal calf serum (FCS) in a 5% CO_2_ incubator at 37 °C. Following stimulation, cells were washed twice with PBS (pH 7.2) and fixed for 15 min with formaldehyde (2% in PBS, pH 7.2). After fixation, cells were washed twice with 0.5% BSA in PBS and permeabilized with PBS containing 0.5% BSA and 0.5% saponin. Cells were then incubated for 15 min at room temperature in the dark with specific fluorochrome-conjugated mAbs, as listed in Table 3. After incubation, cells were washed and analyzed by flow cytometry using a BDLSR II instrument with FACSDiva software. A total of 10^4^ events were acquired for each PB sample, while all events in each AH sample were recorded to ensure comprehensive analysis.

### 4.6. Statistics

Statistical analysis was performed using Origin Software (version 9.9) to evaluate the significance of the differences between groups. Depending on the data distribution, either the Mann–Whitney U test or paired Student’s *t*-test was applied for comparisons. A *p*-value of 0.05 or less was considered statistically significant to assess the relevance of the findings. Results were expressed with the following significance levels: * *p* ≤ 0.05, ** *p* ≤ 0.01, and *** *p* ≤ 0.001.

## 5. Conclusions

Our study showed the presence of significant numbers of lympho-monocytes in the AH of a cohort of patients with RP that were completely absent in the control group. Additionally, there was a significant enrichment of a CD3+CD4-CD8- DN T cell subpopulation, suggesting that an immune response and the consequent intraocular inflammation may be involved in the pathogenesis of RP. This observation may open a clinically relevant therapeutic window for this disorder. 

## Figures and Tables

**Table 1:** Patient characteristics.

	RP Group	Control Group
Total number of patients	N = 12 (N = 14 eyes)	N = 9 (N = 9 eyes)
Median age (years) +/− SD	57.1 (+/−8.2)	77.11 (+/−9.8 years)
Gender (M:F)	6:6	3:6

**Table 2:** List of fluorochrome-conjugated mAbs used for surface markers evaluation.

Antigen	Fluorochrome	Clone	Company
CD16	FITC	3G8	BDBioscience (San Jose, CA, USA)
CD3	Pacific Blue	UCHT1	BDBioscience
CD8	PerCP	SK1	BDBioscience
CD4	PE-Cy7	SK3	eBioscience™ (Waltham, MA, USA)
CD14	APC	MΦP9	BDBioscience
CD19	APC-Cy7	SJ25C1	BDBioscience

**Table 3:** List of fluorochrome-conjugated mAbs used for surface markers and intracellular cytokines evaluation.

Antigen	Fluorochrome	Clone	Company
IFN-γ	FITC	25723.11	BDBioscience
IL-4	PE	3010.211	BDBioscience
IL-17	PerCP-Cy5.5	eBio64DEC17	eBioscience™
CD3	Pacific Blue	UCHT1	BDBioscience
CD8	APC-Cy7	SK1	BDBioscience
CD4	PE-Cy7	SK3	eBioscience™
CD161	APC	DX12	BDBioscience

## Data Availability

The datasets used and analyzed during the current study are available from the corresponding author upon reasonable request. The data that support the findings of this study are available upon request from the corresponding author.
